# Editorial: Advanced functional materials for disease diagnosis, drug delivery and tissue repair

**DOI:** 10.3389/fbioe.2025.1602628

**Published:** 2025-04-11

**Authors:** Liqun Yang, Xianzhi Zhang

**Affiliations:** ^1^ Research Center for Biomedical Materials, Shenyang Key Laboratory of Biomedical Polymers, Engineering Research Center of Ministry of Education for Minimally invasive Gastrointestinal Endoscopic Techniques, Shengjing Hospital of China Medical University, Shenyang, China; ^2^ Department of Biomedical Engineering, Yale University, New Haven, CT, United States

**Keywords:** advanced functional materials, disease diagnosis, sensing, imaging, drug delivery, tissue repair

In recent years, with the rapid advancement of science and technology, advanced functional materials have demonstrated extensive potential in disease diagnosis, drug delivery, and tissue repair (Shi, 2009; Hu et al., 2020; Fenton et al., 2018; Varanko et al., 2020; Whitaker et al., 2021; Christman, 2019). The current Research Topic, Advanced Functional Materials for Disease Diagnosis, Drug Delivery, and Tissue Repair, focuses on the theoretical breakthroughs and multidimensional applications of these emerging materials, exploring how interdisciplinary collaboration drives medical innovation. By integrating materials science, biotechnology, and clinical needs, these studies provide systematic solutions to complex pathological challenges in modern medicine and lay a solid foundation for precision medicine and regenerative medicine.

In the field of diabetes management, significant progress has been achieved in precision diagnostics and chronic wound repair. Guan et al. reported the integration of intelligent nanosensor technology and biosensors, enabling more efficient and non-invasive dynamic glucose monitoring with significantly enhanced sensitivity and specificity (Guan and Zhang). Moreover, Mills et al. demonstrated that dressings coated with multipotent adult progenitor cells (MAPC) accelerated angiogenesis and anti-inflammatory responses, reducing the healing time of chronic diabetic wounds by 40% (Mills et al.). Additionally, Chen et al. developed a synergistic approach using graphene oxide (GO) alginate hydrogels in combination with platelet-rich plasma (Chen et al.), significantly enhancing collagen synthesis and microvascular growth, offering a promising strategy for the precise treatment of complex and irregular wounds.

In the field of cancer diagnosis and treatment, functional materials are revolutionizing the way malignant tumors are diagnosed and treated. For tumor imaging, Zhou et al. highlighted the use of PL002 bimodal contrast agents for precise localization during glioblastoma surgery, substantially improving the efficiency of tumor removal (Zhou et al.). Na et al. summarized major breakthroughs in the use of metallic nanoparticles and carbon-based materials for cerebrovascular imaging, enhancing the ability to conduct early screening for conditions such as aneurysms and strokes (Na et al.). On the therapeutic front, Xu et al. reported that metal-based nanomaterials exhibited synergistic effects in photothermal and immunotherapy (Xu et al.), paving a new pathway for precise breast cancer treatment. Zhao et al. investigated the controlled drug-release capabilities of metal-organic frameworks (MOFs) (Zhao et al.), which have now become a critical tool in treating bone tumors. Ren et al. successfully fabricated SPIO/TP53/PLGA nanobubbles with a diameter of approximately 200 nm and, in combination with low-intensity focused ultrasound, achieved targeted delivery of the TP53 gene, significantly promoting the apoptosis of osteosarcoma cells and providing substantial support for gene-targeted therapies of malignant tumors (Ren et al.). Furthermore, Liu et al. developed a novel sonosensitizer, La_2_(WO_4_)_3_/CuWO_4_ composite material (LC-10), through a two-step hydrothermal method (Liu et al.). When used in a U251 glioblastoma cell model, this composite material inhibited electron-hole (e^−^–h^+^) recombination and generated stronger oxidative reactive oxygen species (ROS), effectively inducing glioblastoma cell apoptosis.

In the field of chronic wound repair, multifunctional smart materials have shown remarkable therapeutic potential. For infectious chronic wounds, Yang et al. developed CS/PVP composite nanofiber membranes with antimicrobial and antioxidant properties (Yang et al.), which not only enhanced the healing efficiency of acute burn wounds but also improved angiogenesis and re-epithelialization. Dai et al. designed shellac microspheres for the sustained release of nonsteroidal anti-inflammatory drugs (e.g., ketoprofen) (Yang et al.), extending bioactivity for up to 3 weeks and effectively reducing systemic side effects of conventional drugs. Regarding antibacterial applications, Zheng et al. created fluoride-releasing nano-zirconia-based composite coatings with long-term fluoride release, high wear resistance, and mechanical stability (Zheng et al.), representing a groundbreaking advancement for dental restorative materials. Tian et al. further demonstrated that multifunctional smart biomaterials hold significant promise for improving wound healing and preventing infections (Tian and Bian).

Repair and engineering of the nervous system have also benefited from the innovative design of functional materials. In spinal cord injury repair, Zhang et al. developed a dual-phase hydrogel scaffold (DPSH) capable of the controlled co-release of neurotrophic factors and Ang-(1–7) (Zhang et al.), effectively reducing local inflammation, promoting neural stem cell proliferation and differentiation, and significantly enhancing the recovery of motor function. In the urological and skeletal systems, the exploration of functional materials also yielded success. Hu et al. proposed that biodegradable ureteral stents (BUS), incorporating antibacterial coating and drug delivery technologies (Hu et al.), can mitigate infections and complications caused by long-term stent implantation, thereby improving patient safety and comfort. Moreover, Cao et al. fabricated PCL-PEG/CS/AST nanofibers (Cao et al.), which induced osteogenic differentiation of bone marrow mesenchymal stem cells (BMSCs), greatly promoting bone repair and functional reconstruction, providing a powerful tool for precision treatment of complex bone diseases.

In conclusion, advanced functional materials, with their multifunctional integration and precise design capabilities, offer multidimensional solutions to complex diseases. Under the impetus of interdisciplinary collaboration in materials science, biology, medicine, and engineering, these research outcomes have enabled dynamic diagnosis, intelligent drug delivery, and tissue regeneration, paving the way for clinical translation in precision medicine. However, several challenges persist, such as optimizing biocompatibility, controlling production costs, and accelerating regulatory approval processes. Looking forward, artificial intelligence may play a critical role in optimizing material properties and designing drug delivery systems, further accelerating the development of smart medical technologies. With continued research progress, multilayered medical technologies involving precise diagnosis, personalized treatment, and tissue repair will gradually emerge in clinical practice, bringing greater hope to patients worldwide and advancing global health to unprecedented heights.
